# Prevalence and Clinical Impact of *BRAF* p.V600E Mutation in Papillary Thyroid Carcinoma

**DOI:** 10.1007/s12022-025-09859-y

**Published:** 2025-04-16

**Authors:** Alexandria Brumfield, Sara Abou Azar, Rachel Nordgren, Ronald N. Cohen, David Sarne, Xavier M. Keutgen, Megan Applewhite, Peter Angelos, Nicole A. Cipriani

**Affiliations:** 1 School of Medicine, Case Western Reserve University, 9501 Euclid Ave, Cleveland, OH 44106 USA; 2 Department of Surgery, The University of Chicago Medicine, 5841 S. Maryland Avenue, MC 6040, Chicago, IL 60637 USA; 3 Department of Public Health Sciences, The University of Chicago, 5841 S. Maryland Avenue, MC 2000, Chicago, IL 60637 USA; 4 Department of Medicine, The University of Chicago Medicine, 5841 S. Maryland Avenue, MC 6040, Chicago, IL 60637 USA; 5 Department of Pathology, The University of Chicago Medicine, 5841 S. Maryland Avenue, MC 6040, Chicago, IL 60637 USA

**Keywords:** Papillary thyroid carcinoma, BRAF, Immunohistochemistry, Recurrence

## Abstract

Identifying risk factors in papillary thyroid carcinoma (PTC) that warrant more aggressive treatment is paramount. Importantly, the prevalence and clinical significance of *BRAF* p.V600E mutation in PTC remain debatable. This study aims to determine the association of *BRAF* p.V600E with demographic and clinicopathologic characteristics, including recurrence. Single institution data from consecutive PTC patients with *BRAF* p.V600E immunohistochemistry and/or molecular testing was collected between 2018 and 2022, including BRAF status, morphologic subtype, TN category, tumor size, nodal disease burden, tumor multifocality, extrathyroidal extension, treatment, follow-up time, loco-regional and distant recurrence, and mortality. This study included 301 patients, 30% male. The majority had *BRAF* p.V600E mutation (78.7%), and *BRAF* p.V600E was associated with morphologic subtype (*p* < 0.001), with 88% of classic subtype PTCs, 38% of PTCs with extensive follicular growth, and 100% of tall cell subtype expressing *BRAF* p.V600E. *BRAF* p.V600E was not associated with tumor size (*p* = 0.696) or nodal disease burden (*p* = 0.962). On multivariate analysis using Cox proportional hazard model, large volume nodal disease burden (HR 3.37, 95%CI 1.49–7.64, *p* = 0.004) and male gender (HR 2.29, 95%CI 1.23–4.26, *p* = 0.009) were significantly associated with recurrence. *BRAF* p.V600E (HR 0.71, 95% CI 0.31–1.65, *p* = 0.4) was not significantly associated with recurrence. In conclusion, presence of *BRAF* p.V600E in the absence of high risk histologic features does not have an impact on PTC recurrence, and thus, its utility in risk stratification is questionable in the setting of other clinicopathologic risk factors.

**Supplementary Information:**

The online version contains supplementary material available at 10.1007/s12022-025-09859-y.

## Introduction

Thyroid follicular cell-derived neoplasms are largely categorized as papillary carcinomas or follicular patterned neoplasms; approximately 85% of thyroid cancers are papillary thyroid carcinoma (PTC) [1]. PTCs frequently harbor *BRAF* p.V600E (or *BRAF* p.V600E-like) mutation, whereas follicular neoplasms harbor *RAS* or (*RAS*-like) mutation [2–4]. *BRAF* p.V600E and mutant *RAS* drive MAPK signaling through distinct molecular mechanisms; mutant *RAS* also drives PI3 K/AKT signaling [2]. PTCs that do not harbor *BRAF* p.V600E often express gene fusions in a mutually exclusive manner, notably receptor tyrosine kinase fusions involving *RET*, *NTRK1/3*, and *ALK*, as well as *BRAF* fusions [2–4].

The reported frequency of *BRAF* p.V600E in PTC varies dramatically, from 27 to 83% in adult PTC to 0–63% in pediatric PTC [5]. Furthermore, prognostic implications of *BRAF* p.V600E remain controversial. Meta-analyses argue that *BRAF* p.V600E predicts lymph node metastasis, but whether *BRAF* p.V600E is associated with PTC subtype, tumor size, loco-regional recurrence, and distant metastasis is not clear [6–11]. The historical diagnostic criteria of PTC subtypes may partially be driving this variation.

PTC has historically been diagnosed based on nuclear features [12]. Subtypes of PTC, including classic, follicular variant, and tall cell, are diagnosed based on cytoarchitectural features. The classic subtype displays a papillary architecture and is infiltrative [12]. The follicular variant is characterized by a predominance of follicular architecture [12] and is often considered less aggressive [13]. However, this variant may encompass both *RAS*-like invasive encapsulated “PTC” and *BRAF* p.V600E-like infiltrative PTC. The tall cell subtype is composed of at least 30% cells that are three times as tall as they are wide[14] and is argued to be more aggressive [13, 15–17]. Importantly, since 2016, a subset of neoplasms with follicular architecture and “PTC-like” nuclei have been separated into noninvasive follicular thyroid neoplasm with papillary-like nuclear features (NIFTP) [18, 19]. These tumors are no longer classified as follicular variants of PTC. Instead, they are considered low-risk neoplasms in the 5 th edition of the World Health Organization Classification of Tumours [20]. If meeting strict criteria, NIFTPs display follicular growth, frequent *RAS* mutations, circumscription, and indolent behavior. This change in classification is clinically significant as neither completion thyroidectomy nor radioactive iodine (RAI) ablation is recommended treatment for NIFTP [21].

The most recent (2015) American Thyroid Association (ATA) guidelines on management of differentiated thyroid carcinoma developed a best estimate Modified Initial Risk Stratification System for recurrence in thyroid carcinomas after initial surgical resection to aid in the post-surgical medical treatment, including RAI if indicated [22]. Tumors are divided into low, intermediate, and high-risk groups for structural local recurrence. The current 2015 ATA guidelines argue that with additional verifying studies, presence of *BRAF* p.V600E could be used to upgrade carcinomas > 1 cm to at least intermediate risk [22]. Whether this risk profile will persist in future guidelines remains to be seen. In addition to *BRAF* p.V600E status, tumor characteristics such as tall cell subtype, tumor size, and volume of nodal involvement are also considered when determining treatment. Tumor size is argued to be a risk factor for recurrence [23–25], and recurrence is argued to be more frequent in PTCs with a higher number or larger size of lymph node metastases [26–28].

In light of changes in classification and the importance of *BRAF* p.V600E in recurrence risk stratification, this study sought to determine the prevalence of *BRAF* p.V600E in consecutive PTCs diagnosed at the University of Chicago Medical Center, ensuring NIFTPs were excluded from analysis. *BRAF* p.V600E immunohistochemical stain (IHC) (VE1 clone), which is highly sensitive and specific for *BRAF* p.V600E mutation [29], was utilized. This study also aimed to correlate *BRAF* p.V600E mutational status with characteristics including PTC subtype, T and N category, tumor size, nodal disease burden, loco-regional recurrence, and distant metastasis.

## Methods

### Patient and Tumor Inclusion

**Fig. 1:**
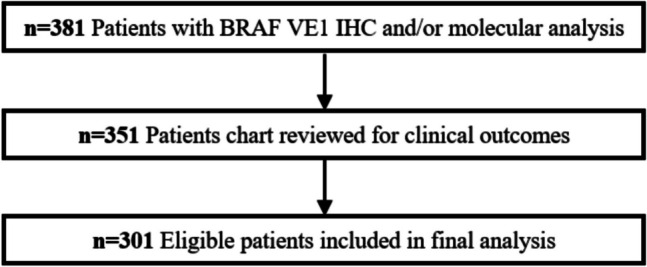
Flow diagram of patient inclusion. 381 PTC patients with BRAF VE1 IHC and/or molecular analysis performed between 2018 and 2023 were identified. Clinical outcomes were determined for patients with molecular analysis or IHC performed between 2018 and 2022. After excluding patients without a tumor size, no post-surgery follow-up, and tumors > 1 cm not of classic, with extensive follicular growth, or tall cell subtype, the final analysis included 301 patients

This retrospective study was conducted with IRB approval. The surgical pathology archives at the University of Chicago were searched for patients with PTC who had *BRAF* p.V600E IHC (VE1 clone) or molecular analysis performed between June 1, 2018, and May 1, 2023. During this time period, BRAF VE1 IHC was performed on all newly diagnosed or recurrent PTCs (if mutational status was not otherwise known). NIFTPs were not included. A total of 381 patients were identified. Clinical outcomes were available for 351 of the 381 patients, which included all patients with molecular analysis between June 1, 2018, and May 1, 2023, and patients with IHC analysis performed between June 1, 2018, and December 31, 2022. This restriction in dates was to ensure patients had the possibility of 6 months of follow-up. Patients were excluded if they had (i) an unavailable tumor size, (ii) no post-surgery follow-up, or (iii) a tumor > 1 cm not classified as classic, with extensive follicular growth, or tall cell subtype. A total of 301 patients were included in the final analysis (Fig. 1).

### Data Collection

Demographic, clinical, and pathologic data from surgical pathology reports were collected from the electronic medical record (EMR). All surgical pathology diagnoses were made by University of Chicago pathologists. Demographic and clinicopathologic data collected for patients included: BRAF VE1 IHC and *BRAF* p.V600E molecular status, age at initial thyroid surgery, sex, race, PTC subtype, T and N category at initial surgery, tumor size, number of involved nodes, size of nodal deposit, tumor multifocality, extrathyroidal extension (grossly into at least skeletal/strap muscle), treatment, follow-up time, initial loco-regional recurrence, initial distant metastasis, and death.

BRAF IHC and molecular status were deemed negative or positive. One patient with both BRAF IHC and molecular data was false negative on IHC (Supplemental Fig. 1); thus, in analyses, this patient was deemed *BRAF* p.V600E positive. Using surgical pathology reports, PTCs were subclassified as classic, with extensive follicular growth, or tall cell subtype. In light of the challenges in nomenclature and morphologic variability in “follicular variant of PTC,” only cases of classic PTC with prominent follicular architecture/extensive follicular growth (EFG) were included and were classified as “PTC-EFG” if demonstrating an infiltrative growth pattern and > 75% follicular growth. Cases meeting criteria for the *RAS*-like “invasive encapsulated follicular variant of PTC” were excluded. Patients with other rare PTC subtypes (including oncocytic, cribriform morular, solid/trabecular, hobnail, columnar, and diffuse sclerosing) and PTCs with high grade features according to WHO 5 th edition definition (including necrosis, increased mitoses, poorly differentiated morphology, or anaplastic morphology) were excluded. Morphologic subtype was not mentioned for 28 PTCs ≤ 1 cm, formerly known as microcarcinomas and not requiring subtyping; in these cases, PTC subtype was denoted as “not available.” T and N category at initial surgery was assigned based on AJCC 8 guidelines. Primary tumor size was defined in the largest dimension and was divided into 4 subgroups: ≤ 1 cm, > 1 and ≤ 2 cm, > 2 and ≤ 4 cm, and > 4 cm. Lymph node (LN) disease burden on final pathology was classified into none (LNs negative or no LNs harvested), small volume (≤ 5 involved LNs all ≤ 0.2 cm in greatest dimension or psammoma bodies only), and large volume (> 5 involved LNs and/or > 0.2 cm in greatest dimension). Multifocality was defined as more than one tumor in either the same or the opposite thyroid lobe. For multifocal tumors, BRAF mutational status was assigned based on the BRAF mutational status of the largest tumor. Treatment was classified as total thyroidectomy (TT), TT, and postoperative RAI ablation therapy, other, which included thyroid lobectomy and sub-total thyroidectomy, or none, which included 4 patients that were diagnosed with distant metastases prior to initial thyroid surgery. End-point was considered recurrence, death, or time of last follow-up, whichever occurred first. Initial treatment was considered to have been completed before date of end-point (specifically, RAI administered following recurrence was not considered part of initial therapy). Follow-up time was determined from time of initial thyroid surgery to last known thyroid-specific clinical encounter. Local recurrence was defined as PTC recurrence in the thyroid bed or regional lymph nodes and distant metastasis was defined as PTC in visceral sites such as the lung, bone, or brain. Loco-regional recurrence or distant metastasis was confirmed by biopsy/resection or by diagnostic radiographic findings, which were used as date of loco-regional recurrence or distant metastasis. In analyses, recurrence within 10 years includes both loco-regional recurrence and distant metastasis diagnosed after initial thyroid surgery. Of note, although patients had IHC and/or molecular testing between 2018 and 2023, some patients had their initial thyroid surgery prior to 2018. Time to recurrence was calculated from date of initial thyroid surgery.

### BRAF Immunohistochemistry and Molecular Analysis

BRAF VE1 immunohistochemical (IHC) stain was prospectively performed on PTCs starting in November 2018 as part of the routine prognostic workup: Abcam, mouse monoclonal, clone VE1 (catalogue # ab228461), 1:50 titer, 25-min incubation, performed on Leica Bond III instrument, using online antigen retrieval (Bond Epitope Retrieval Solution 2), and visualized using Leica Refine DAB Detection Kit. BRAF was not performed on incidental intrathyroidal nonmetastatic PTCs ≤ 0.5 cm. All other cases were tested. Select cases were subject to molecular analysis, as part of clinical decision-making. Methods of sequencing included UChicago Medicine OncoPlus DNA [30] and RNA [31] sequencing panel on formalin-fixed, paraffin embedded (FFPE) tissue or fine needle aspirate (FNA) or reports from outside analysis (ThyGeNEXT, Afirma, ThyroSeq, Tempus, Foundation One, etc.).

### Statistical Analysis

R 4.2.3 (R Core Team, 2023, a language and environment for statistical computer, R Foundation for Statistical Computing. Vienna, Austria) was used for the statistical analysis, including packages ggplot2, survival, and survminer [32–34]. Descriptive analyses were performed for demographics and tumor characteristics, and the chi-squared test was used to compare categorical variables. Kaplan–Meier curves were generated for survival. Univariate and multivariate analyses using Cox regression models were used for recurrence analysis. Proportional hazards assumptions were upheld with a *p* > 0.05 for all variables in the univariate and multivariate models; survival::cox.zph was used to test proportional hazards. Four variables rejected proportional hazards and were excluded from regression models: tumor size, T category, treatment, and RAI. Confounding was evaluated by comparing coefficients between univariate and multivariate regression results. Variable selection was performed to remove confounding variables including N category and number of nodes.

## Results

### Study Population

**Fig. 2:**
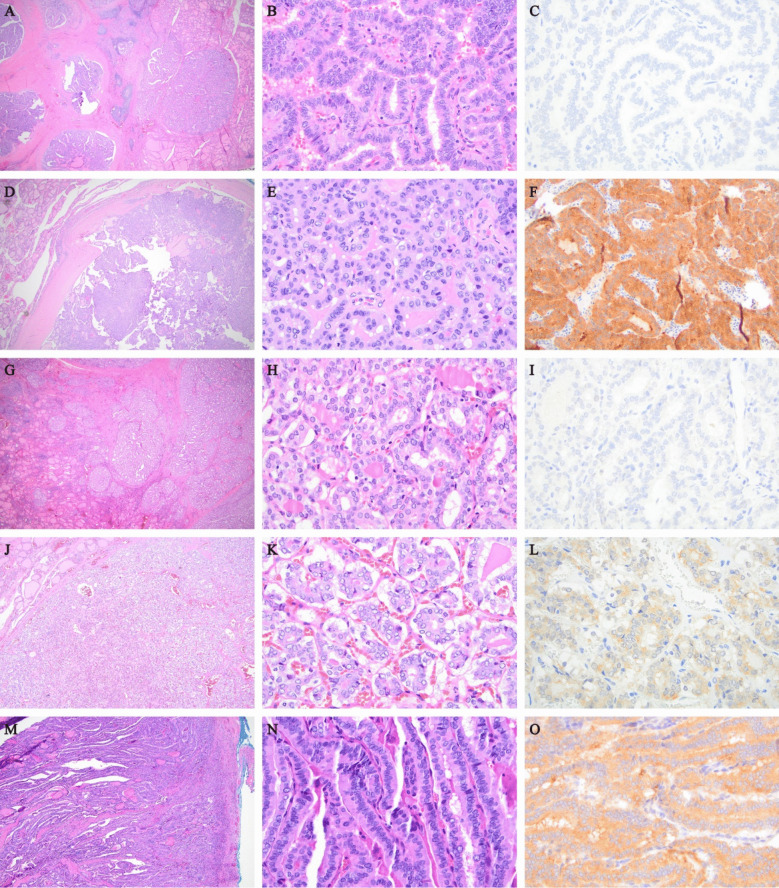
Hematoxylin and eosin (H&E) stain of a *BRAF* p.V600E negative classic subtype (**A**–**C**) papillary thyroid carcinoma (PTC) shows papillary morphology on low-power (**A**) and high-power (**B**) views; corresponding *BRAF* p.V600E immunohistochemistry (IHC) (VE1 clone) shows an absence of cytoplasmic staining (**C**). H&E stain of a *BRAF* p.V600E positive classic subtype (**D**–**F**) PTC on low-power (**D**) and high-power (**E**) views; corresponding *BRAF* p.V600E IHC shows cytoplasmic staining (**F**). H&E stain of a *BRAF* p.V600E negative PTC with extensive follicular growth (EFG) (**G**–**I**), harboring *ETV6::NTRK3* fusion, shows follicular morphology on low-power (**G**) and high-power (**H**) views; corresponding *BRAF* p.V600E IHC shown in (**I**). H&E stain of a *BRAF* p.V600E positive PTC with EFG (**J**–**L**) on low-power (**J**) and high-power (**K**) views; corresponding *BRAF* p.V600E IHC is shown in (**L**). H&E stain of a *BRAF* p.V600E positive tall cell subtype (**M**–**O**) PTC shows elongated papillary structures and tumor cells at least three times as tall as they are wide on low-power (**M**) and high-power (**N**) views; corresponding *BRAF* p.V600E IHC shown in (**O**)

The final study population included 301 patients with PTC (Fig. 1) of which 262 had *BRAF* p.V600E IHC (VE1 clone) analysis only, 14 had molecular analysis only, and 25 had both *BRAF* p.V600E IHC and molecular analysis. Of the 25 with IHC and molecular analysis, there was one false negative on IHC (Supplemental Fig. 1); *BRAF* p.V600E IHC sensitivity was 94% and specificity was 100%. Examples of negative and positive BRAF VE1 staining in PTC are shown in Fig. 2.

Of the 301 patients, 78.7% were *BRAF* p.V600E positive (*n* = 237) and 21.3% were negative (*n* = 64). In only 4 patients with multifocal disease, the largest tumor was *BRAF* negative while a smaller focus was *BRAF* positive; in 5 patients, the largest tumor was *BRAF* positive while a smaller focus was *BRAF* negative. Average age was 48.1 years. The majority of patients were female (70%) and white (66%). No statistical difference in age, sex, or race was observed between *BRAF* p.V600E positive and negative patients. The demographics and clinicopathological characteristics of all patients, as well as patients with and without BRAF p.V600E mutation, are listed in Table 1.

**Table 1:** Demographics and clinicopathological characteristics, stratified by *BRAF* p.V600E status

	BRAF negative (*n* = 64)	BRAF positive (*n* = 237)	All (*n* = 301)	*p*-value
**Age**	44.7 (29.6, 60.3)	48.9 (34.1, 60.6)	48.1 (33.7, 60.6)	0.253
**Sex (male)**	18 (28%)	72 (30%)	90 (30%)	0.727
**Race**				0.374
Other or unknown	13 (20%)	65 (27%)	78 (26%)	
Black	7 (11%)	17 (7%)	24 (8%)	
White	44 (69%)	155 (65%)	199 (66%)	
**Subtype**				** < 0.001**
Classic	23 (36%)	162 (68%)	185 (61%)	
EFG*	33 (52%)	20 (8%)	53 (18%)	
Tall	0 (0%)	35 (15%)	35 (12%)	
Not available	8 (12%)	20 (8%)	28 (9%)	
**T category**				0.65
T1	36 (56%)	148 (62%)	184 (61%)	
T2	17 (27%)	56 (24%)	73 (24%)	
T3 or T4	11 (17%)	33 (14%)	44 (15%)	
**N category**				0.878
N0	15 (23%)	60 (25%)	75 (25%)	
N1	34 (53%)	128 (54%)	162 (54%)	
Nunk**	15 (23%)	49 (21%)	64 (21%)	
**Size in cm**				0.696
≤ 1 cm	19 (30%)	64 (27%)	83 (28%)	
> 1 and ≤ 2 cm	20 (31%)	90 (38%)	110 (37%)	
> 2 and ≤ 4 cm	18 (28%)	65 (27%)	83 (28%)	
> 4 cm	7 (11%)	18 (8%)	25 (8%)	
**Nodal disease burden on pathology**				0.962
None	30 (47%)	109 (46%)	139 (46%)	
Small volume***	6 (9%)	25 (11%)	31 (10%)	
Large volume****	28 (44%)	103 (43%)	131 (44%)	
**Multifocal**	27 (42%)	115 (49%)	142 (47%)	0.368
**Extrathyroidal extension**	4 (6%)	22 (9%)	26 (9%)	0.443
**Treatment**				0.105
None	0 (0%)	4 (2%)	4 (1%)	
Other*****	12 (19%)	57 (24%)	69 (23%)	
TT	23 (36%)	52 (22%)	75 (25%)	
TT + RAI	29 (45%)	124 (52%)	153 (51%)	
**Follow-up (months)**	19.5 (6.7, 33.3)	24.5 (13.3, 39.2)	23.4 (11.9, 37.2)	**0.031**
**Recurrence within 10 years**	8 (12%)	36 (15%)	44 (15%)	0.589
**Distant metastasis**	1 (2%)	9 (4%)	10 (3%)	0.376
**Death**	1 (2%)	2 (1%)	3 (1%)	0.608

*EFG refers to PTC with extensive follicular growth

**Nunk refers to N unknown

***Small volume refers to N1a/N1b tumor with nodal disease burden on final pathology ≤ 5 LNs and ≤ 0.2 cm

****Large volume refers to N1a/N1b tumor with nodal disease burden on final pathology > 5 LNs and/or > 0.2 cm

*****Other includes thyroid lobectomy and sub-total thyroidectomy

*p*-values in bold are statistically significant

### PTC Subtype

The classic subtype of PTC was most common (*n* = 185, 61%). Fifty-three patients had PTC with extensive follicular growth (EFG) (18%) and 35 patients had tall cell subtype (12%). Subtype was significantly different between *BRAF* p.V600E positive and negative tumors (*p* < 0.001) (Table 1), with 88% of classic (*n* = 162) and 100% of tall cell subtype tumors (*n* = 35) being *BRAF* p.V600E positive, in contrast to 38% of EFG (*n* = 20) (Table 2). The demographics and clinicopathological characteristics of patients stratified by PTC subtype are listed in Table 2 and Supplemental Table 1. Examples of classic, with extensive follicular growth, and tall cell morphology in *BRAF* p.V600E IHC negative and positive PTC tumors are shown in Fig. 2.

**Table 2:** Demographics and clinicopathological characteristics, stratified by tumor subtype

	Classic (*n* = 185)	EFG (*n* = 53)	Tall (*n* = 35)	Not available (*n* = 28)	*p*-value
**Age**	44.3 (33.1, 58.9)	54.7 (36.2, 66.5)	54.2 (43.4, 61.2)	48.1 (35.1, 54.5)	0.142
**Sex (male)**	55 (30%)	13 (25%)	12 (34%)	10 (36%)	0.681
**BRAF positive**	162 (88%)	20 (38%)	35 (100%)	20 (71%)	** < 0.001**
**T category**					** < 0.001**
T1	112 (61%)	32 (60%)	12 (34%)	28 (100%)	
T2	47 (25%)	14 (26%)	12 (34%)	0 (0%)	
T3 or T4	26 (14%)	7 (13%)	11 (31%)	0 (0%)	
**N category**					**0.006**
N0	38 (21%)	15 (28%)	10 (29%)	12 (43%)	
N1	112 (61%)	24 (45%)	20 (57%)	6 (21%)	
Nunk	35 (19%)	14 (26%)	5 (14%)	10 (36%)	
**Treatment**					** < 0.001**
None	3 (2%)	1 (2%)	0 (0%)	0 (0%)	
Other	38 (21%)	13 (25%)	4 (11%)	14 (50%)	
TT	48 (26%)	14 (26%)	1 (3%)	12 (43%)	
TT + RAI	96 (52%)	25 (47%)	30 (86%)	2 (7%)	
**Follow-up (months)**	22.2 (10.2, 36.6)	27.2 (14.3, 41.5)	28.8 (18.6, 48.4)	22.3 (2.4, 37.3)	**0.018**
**Recurrence within 10 years**	23 (12%)	8 (15%)	12 (34%)	1 (4%)	**0.003**
**Distant metastases**	5 (3%)	2 (4%)	3 (9%)	0 (0%)	0.239
**Death**	2 (1%)	0 (0%)	1 (3%)	0 (0%)	0.561

*p*-values in bold are statistically significant

### T and N Category

One hundred eighty-four (61%) tumors were categorized as T1, 73 (24%) as T2, and 44 (15%) as T3 or T4, and 75 (25%) tumors were categorized as N0 and 162 (54%) as N1. No statistical difference was observed between T or N category based on *BRAF* p.V600E positivity (Table 1). When stratified by PTC subtype, 31% of tall cell tumors were T3 or T4 category, compared to 14% of classic and 13% of EFG tumors (*p* < 0.001) (Table 2).

### Tumor Size

Primary tumors ≤ 1 cm were found in 83 patients (28%), while 110 tumors (37%) were > 1 and ≤ 2 cm, 83 (28%) were > 2 and ≤ 4 cm, and 25 (8%) were > 4 cm. *BRAF* p.V600E positive and negative tumors were not significantly different based on tumor size (Table 1), with *BRAF* p.V600E positivity in 77% of tumors ≤ 1 cm, 82% of tumors > 1 and ≤ 2 cm, 78% of tumors > 2 and ≤ 4 cm, and 72% of tumors > 4 cm (Table 3). The demographics and clinicopathological characteristics of patients stratified by tumor size are listed in Table 3 and Supplemental Table 2.

**Table 3:** Demographics and clinicopathological characteristics, stratified by primary tumor size in cm

	≤ 1 cm (*n* = 83)	> 1 and ≤ 2 cm (*n* = 110)	> 2 and ≤ 4 cm (*n* = 83)	> 4 cm (*n* = 25)	*p*-value
**Age**	49.6 (34.4, 59.4)	51.4 (34.8, 65.9)	43.1 (32.7, 56.2)	45.3 (33.9, 57.4)	0.118
**Sex (male)**	24 (29%)	29 (26%)	24 (29%)	13 (52%)	0.087
**BRAF positive**	64 (77%)	90 (82%)	65 (78%)	18 (72%)	0.696
**T category**					** < 0.001**
T1	83 (100%)	101 (92%)	0 (0%)	0 (0%)	
T2	0 (0%)	0 (0%)	73 (88%)	0 (0%)	
T3 or T4	0 (0%)	9 (8%)	10 (12%)	25 (100%)	
**N category**					** < 0.001**
N0	31 (37%)	25 (23%)	18 (22%)	1 (4%)	
N1	32 (39%)	55 (50%)	54 (65%)	21 (84%)	
Nunk	20 (24%)	30 (27%)	11 (13%)	3 (12%)	
**Treatment**					** < 0.001**
None	0 (0%)	2 (2%)	1 (1%)	1 (4%)	
Other	34 (41%)	26 (24%)	9 (11%)	0 (0%)	
TT	27 (33%)	25 (23%)	18 (22%)	5 (20%)	
TT + RAI	22 (27%)	57 (52%)	55 (66%)	19 (76%)	
**Follow-up (months)**	20.4 (7.8, 33.6)	23.5 (11.6, 37.0)	24.4 (13.7, 40.3)	30.5 (17.3, 49.2)	**0.013**
**Recurrence within 10 years**	4 (5%)	16 (15%)	16 (19%)	8 (32%)	**0.003**
**Distant metastasis**	1 (1%)	2 (2%)	2 (2%)	5 (20%)	** < 0.001**
**Death**	0 (0%)	0 (0%)	1 (1%)	2 (8%)	**0.002**

*p*-values in bold are statistically significant

### Nodal Disease Burden

Of patients with evidence of nodal disease on final pathology, 31 patients (10%) had small volume, and 131 (44%) had large volume nodal involvement. There was no statistical difference in nodal disease burden based on *BRAF* p.V600E positivity (Table 1), with *BRAF* p.V600E positivity in 78% of tumors without nodal metastasis, 81% of tumors with small volume, and 79% of tumors with large volume nodal disease burden (Table 4). The demographics and clinicopathological characteristics of patients stratified by nodal disease burden are listed in Table 4 and Supplemental Table 3.

**Table 4:** Demographics and clinicopathological characteristics, stratified by nodal disease burden on final pathology

	Neither (*n* = 139)	Small volume (*n* = 31)	Large volume (*n* = 131)	*p*-value
**Age**	51.8 (36.6, 65.1)	43.4 (29.6, 57.0)	44.7 (30.6, 56.0)	**0.001**
**Sex (male)**	37 (27%)	6 (19%)	47 (36%)	0.101
**BRAF positive**	109 (78%)	25 (81%)	103 (79%)	0.962
**T category**				** < 0.001**
T1	103 (74%)	18 (58%)	63 (48%)	
T2	27 (19%)	9 (29%)	37 (28%)	
T3 or T4	9 (6%)	4 (13%)	31 (24%)	
**N category**				** < 0.001**
N0	75 (54%)	0 (0%)	0 (0%)	
N1	0 (0%)	31 (100%)	131 (100%)	
Nunk	64 (46%)	0 (0%)	0 (0%)	
**Treatment**				** < 0.001**
None	1 (1%)	0 (0%)	3 (2%)	
Other	52 (37%)	8 (26%)	9 (7%)	
TT	50 (36%)	8 (26%)	17 (13%)	
TT + RAI	36 (26%)	15 (48%)	102 (78%)	
**Follow-up (months)**	21.9 (7.2, 34.6)	27.9 (13.6, 36.1)	26.1 (13.3, 39.8)	0.52
**Recurrence within 10 years**	8 (6%)	2 (6%)	34 (26%)	** < 0.001**
**Distant metastasis**	2 (1%)	0 (0%)	8 (6%)	0.056
**Death**	1 (1%)	0 (0%)	2 (2%)	0.673

*p*-values in bold are statistically significant

### Multifocality and Extrathyroidal Extension

**Fig. 3:**
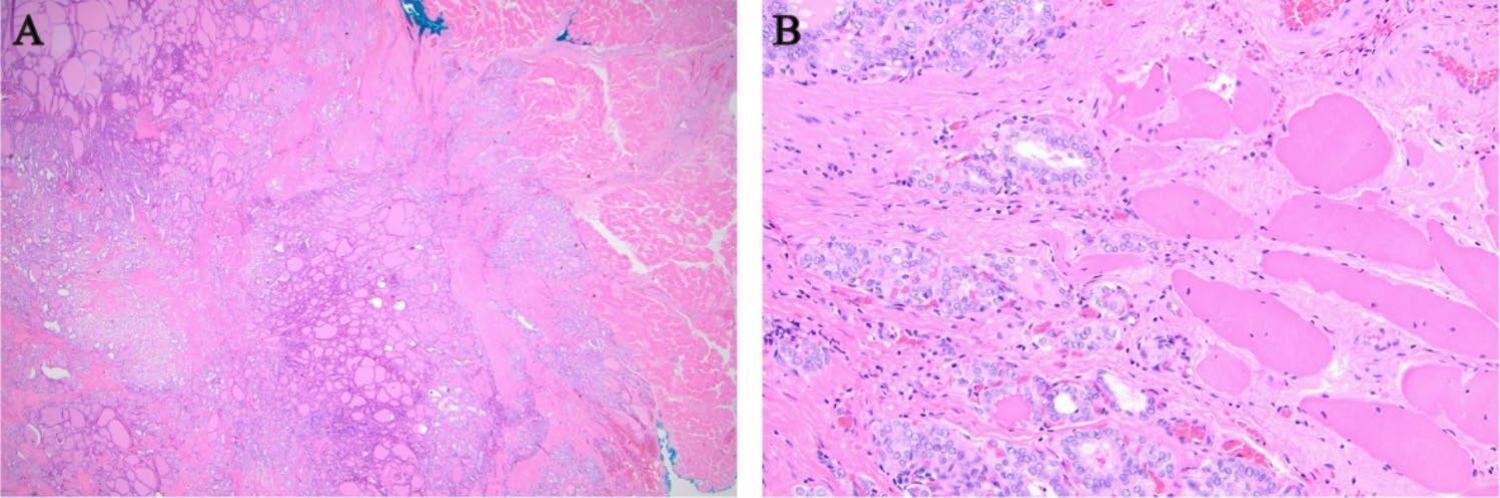
Hematoxylin and eosin (H&E) stains of an example of a papillary thyroid carcinoma (PTC) with extrathyroidal extension (ETE) into skeletal muscle on low-power (**A**) and high-power (**B**) views

Forty-seven percent of patients (*n* = 142) had evidence of multifocal disease, and 9% of patients (*n* = 26) had extrathyroidal extension (ETE) on final pathology. No statistical differences in multifocality and ETE were found between *BRAF* p.V600E positive and negative tumors, although there was a trend toward ETE in *BRAF* p.V600E positive patients (9% vs. 6%) (Table 1). An example of PTC with ETE into skeletal muscle is shown in Fig. 3.

### Treatment

In terms of therapeutic interventions performed before end-point, 25% of patients had total thyroidectomy (TT) (*n* = 75), 51% had TT followed by postoperative RAI (*n* = 153), and 23% (*n* = 69) had other procedures including thyroid lobectomy. Distant metastases were found in 4 patients prior to initial thyroid surgery (1%) and thus were considered to have had no treatment. Although not statistically significant, 52% of patients with *BRAF* p.V600E positivity had TT followed by RAI compared to 45% of patients that were *BRAF* p.V600E negative (Table 1).

When examining treatment between PTC subtypes before end-point, 86% of patients with tall cell PTC had TT followed by RAI, compared to 52% with classic and 47% with EFG (*p* < 0.001) (Table 2). When stratified by tumor size, 27% of tumors ≤ 1 cm, 52% of tumors > 1 and ≤ 2 cm, 66% of tumors > 2 and ≤ 4 cm, and 76% of tumors > 4 cm were treated with TT followed by RAI before end-point (*p* < 0.001) (Table 3). When stratified by nodal disease burden, 26% of patients without nodal disease burden, 48% with small volume, and 78% with large volume nodal disease burden were treated with TT followed by RAI before end-point (*p* < 0.001) (Table 4). Of note, many patients with tumors < 1 cm were N1a/N1b with large volume nodal disease burden, possibly explaining the rates of RAI therapy in these small tumors.

### Recurrence

**Fig. 4:**
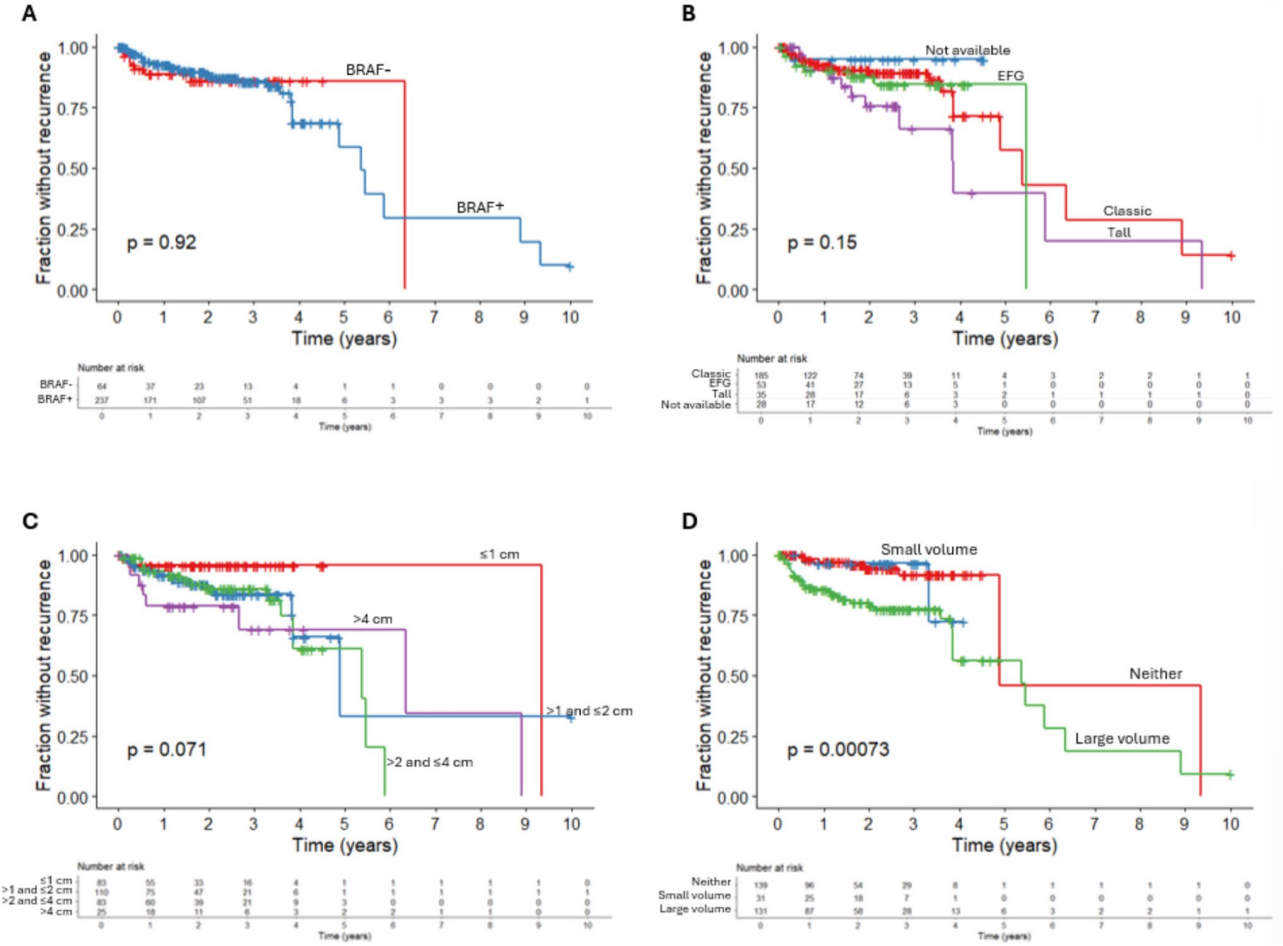
**A** Kaplan–Meier (KM) curve representing recurrence over time for patients with *BRAF* p.V600E positivity on immunohistochemistry or molecular testing vs. patients with *BRAF* p.V600E negativity, *p* = 0.92. **B** KM curve representing recurrence over time stratified by tumor subtype, *p* = 0.15. **C** KM curve representing recurrence over time stratified by primary tumor size, *p* = 0.071. **D** KM curve representing recurrence over time stratified by nodal disease burden on pathology, *p* = 0.00073

Clinical outcomes in the 301 patients were examined; median follow-up was 23.4 months (IQR 11.9, 37.2). Fifteen percent of patients (*n* = 44) had evidence of recurrence within 10 years on imaging and/or biopsy, of which 7 patients had both loco-regional and distant metastasis and the majority had loco-regional recurrence only (*n* = 37). Distant metastases were noted in 3% of patients (*n* = 10) and mortality rate was 1% at study end date (*n* = 3). Follow-up time was longer in *BRAF* p.V600E positive patients at 24.5 months (IQR 13.3, 39.2) compared to 19.5 months (IQR 6.7, 33.3) in *BRAF* p.V600E negative patients (*p* = 0.031). However, no statistical differences in recurrence, distant metastasis, or death were observed between *BRAF* p.V600E positive and negative patients (Table 1). A Kaplan–Meier (KM) curve for recurrence within 10 years showed no statistical difference when stratified by *BRAF* p.V600E positivity (*p* = 0.92) (Fig. 4A). In tumors > 1 cm, no statistical difference in loco-regional recurrence, distant metastasis, or death within 10 years were observed between *BRAF* p.V600E positive and negative patients (Supplemental Table 4). In tumors > 2 and ≤ 4 cm, no statistical difference in loco-regional recurrence, distant metastasis, or death within 10 years were observed, although there was a trend toward increased loco-regional recurrence in *BRAF* p.V600E positive patients (22%) compared to *BRAF* p.V600E negative patients (11%) (Supplemental Table 5).

**Table 5:** Cox proportional hazard model for recurrence: univariate analysis

Characteristic	*N*	HR*	95% CI*	*p*-value
**Age**	301	1.01	0.99, 1.03	0.2
**Sex (male)**	301	2.59	1.43, 4.70	**0.002**
**Race (white)**	301	1.53	0.73, 3.20	0.2
**BRAF positive**	301	0.96	0.44, 2.08	> 0.9
**Subtype**	301			0.15
Classic		—	—	
EFG		1.17	0.52, 2.62	
Tall		1.94	0.95, 3.95	
Not available		0.34	0.05, 2.50	
**N category**	301			**0.004**
None		—	—	
N0		1.23	0.29, 5.18	
N1		3.7	1.13, 12.1	
**Nodal disease burden on pathology**	301			** < 0.001**
None		—	—	
Small volume		1.11	0.24, 5.26	
Large volume		3.74	1.72, 8.13	
**Number of nodes**	301	1.05	1.03, 1.07	** < 0.001**
**Multifocal**	301	1.91	1.03, 3.54	**0.038**
**Extrathyroidal extension**	301	3.29	1.60, 6.75	**0.004**

*HR hazard ratio, *CI* confidence interval

*p*-values in bold are statistically significant

Recurrence was also compared among PTCs stratified by subtype, tumor size, and nodal disease burden. Recurrence was significantly different in the tall subtype (34%) vs. classic (12%) and EFG (15%), with a *p*-value of 0.003 (Table 2). Recurrence was higher among the > 4 cm group compared to smaller tumors (32% vs. 19% for > 2 and ≤ 4 cm, 15% for > 1 and ≤ 2 cm, and 5% for ≤ 1 cm, *p* = 0.003), as well as distant metastasis (*p* < 0.001) and death (*p* = 0.002) (Table 3). Recurrence was also higher in patients who had large volume nodal burden than those with small volume or no nodal disease (26% vs. 6% and 6%, respectively, *p* < 0.001) (Table 4). Kaplan–Meier (KM) curves for recurrence within 10 years show a trend toward statistical difference when stratified by PTC subtype (*p* = 0.15) (Fig. 4B) and tumor size (*p* = 0.071) (Fig. 4C) and a statistical difference when stratified by nodal disease burden (*p* = 0.00073) (Fig. 4D).

On univariate analysis using Cox proportional hazard model for recurrence, *BRAF* p.V600E positivity was not significantly associated with recurrence (*p* > 0.9). However, male sex (*p* = 0.002), higher N category (*p* = 0.004), larger nodal disease burden on pathology (*p* < 0.001), number of involved nodes (*p* < 0.001), multifocality (*p* = 0.038), and extrathyroidal extension (*p* = 0.004) were associated with higher risk of recurrence. Although not statistically significant, tall cell subtype compared to classic trended toward higher risk of recurrence (*p* = 0.15). The univariate analysis for all characteristics using Cox proportional hazard model for recurrence is shown in Table 5.

On multivariate analysis with variable selection performed to remove extraneous variables (see methods), *BRAF* positivity (HR 0.71, 95% 0.31–1.65, *p* = 0.4) was not significantly associated with recurrence. However, male sex (HR 2.29, 95%CI 1.23––4.26, *p* = 0.009) and larger volume of nodal disease burden on pathology (HR 3.37, 95%CI 1.49–7.64, *p* = 0.004) remained significantly associated with higher risk of recurrence. While not significant, tall cell subtype (HR 2.08, 95%CI 0.98–4.41, *p* = 0.055), multifocal disease (HR 1.92, 95%CI 0.99–3.71, *p* = 0.053), and extrathyroidal extension (HR 2.09, 95%CI 0.98–4.43, *p* = 0.056) trended toward worse outcome. The multivariate analysis using Cox proportional hazard model for recurrence is shown in Table 6.

**Table 6:** Cox proportional hazard model for recurrence: multivariate analysis

Characteristic	HR*	95% CI*	*p*-value
**Sex (male)**	2.29	1.23, 4.26	**0.009**
**BRAF positive**	0.71	0.31, 1.65	0.4
**Subtype**			
Classic	—	—	
EFG	1.28	0.53, 3.07	0.6
Tall	2.08	0.98, 4.41	0.055
Not available	0.56	0.07, 4.39	0.6
**Nodal disease burden on pathology**			
None	—	—	
Small volume	1.31	0.26, 6.50	0.7
Large volume	3.37	1.49, 7.64	**0.004**
**Multifocal**	1.92	0.99, 3.71	0.053
**Extrathyroidal extension**	2.09	0.98, 4.43	0.056

*HR hazard ratio, *CI* confidence interval

*p*-values in bold are statistically significant

## Discussion

The reported frequency of *BRAF* p.V600E expression in PTCs is highly variable, 27–83% in adults [5]. In this study, 79% of PTCs harbor *BRAF* p.V600E mutation (Table 1). Specific exclusion of NIFTPs likely explains the relatively high *BRAF* p.V600E-mutated proportion compared to most other published studies, in which exclusion of the *RAS*-like tumors (NIFTP, noninvasive follicular variant of PTC, invasive encapsulated follicular variant of PTC) has not been uniform. In this patient population, *BRAF* p.V600E was only significantly associated with PTC subtype and follow-up time, but not with TN category, tumor size, nodal disease burden, tumor multifocality, extrathyroidal extension, treatment, recurrence, or mortality (Table 1). Both tall cell and classic PTCs highly expressed *BRAF* p.V600E, at 100% and 88% respectively, and a smaller percentage of EFG cases expressed *BRAF* p.V600E at 38% (Table 2). These findings are in line with trends reported in published studies [7, 35, 36]. Follow-up time post initial thyroid surgery was longer in *BRAF* p.V600E positive patients (25 vs. 20 months), possibly because clinical teams choose to follow *BRAF* p.V600E patients more closely. On multivariate analysis, *BRAF* p.V600E positivity was not associated with loco-regional recurrence or distant metastasis (Table 6). Of note, studies argue that *BRAF* p.V600E does not predict clinical outcomes in tumors ≤ 1 cm, formerly known as microcarcinomas [37–39]. Thus, we also examined clinical outcomes when tumors ≤ 1 cm were removed from the data set and found no statistical difference in loco-regional recurrence, distant metastasis, or death in tumors > 1 cm based on *BRAF* p.V600E status (Supplemental Table 4). Therefore, small size is not contributing to the lack of difference in clinical outcomes based on BRAF p.V600E status. Furthermore, there was not a significant difference in loco-regional recurrence, distant metastasis, or death in tumors > 2 and ≤ 4 cm based on *BRAF* p. V600E status (Supplemental Table 5). The prognostic value of *BRAF* p.V600E is highly variable in the literature, with some studies reporting no significance [11, 40–46] and others reporting increased recurrence and worse prognosis [6, 8, 35, 47, 48]. Interestingly, studies that report a significant association between BRAF p.V600E and aggressive clinicopathologic features of PTC have low BRAF p.V600E expression rates (45–49%) [6, 47, 48]. Conversely, studies that fail to demonstrate an association have higher BRAF p.V600E expression rates (70–87%) [40, 44–46], suggesting studies with lower BRAF p.V600E expression rates may include cases of invasive encapsulated or noninvasive encapsulated FV-PTC, many of which are currently classified in the non-malignant, low-risk neoplasm category of NIFTPs. Overall, this and other studies’ findings argue that clinicopathological factors other than *BRAF* p.V600E expression have a stronger effect on recurrence (Table 6). However, the 2015 ATA guidelines suggest upgrading tumors > 1 cm with *BRAF* p.V600E to intermediate risk of recurrence [22]. The results of this study call into question this recommendation. Whether this risk profile will persist in upcoming ATA guidelines remains to be seen.

Several studies argue that tall cell PTCs have a more aggressive behavior than classic PTC [13, 15–17]. ATA guidelines deem tall cell morphology an aggressive histology and upgrades tall cell tumors to at least intermediate risk [22]. Thus, this study sought to determine if morphologic subtype was a risk factor for recurrence. On univariate analysis, tall cell PTCs were 1.94 times more likely to recur than classic PTCs (*p* = 0.15) (Table 5). On multivariate analysis, although not significant, tall cell subtype conferred a 2.08 times higher risk of recurrence (*p* = 0.055) (Table 6). While cases with high grade histologic features were excluded, the possibility that tall cell tumors harbored aggressive genetics (*TERT* promoter or *TP53* mutation) could not be excluded, as molecular testing was not uniformly performed. Additional studies to include greater numbers of tall cell tumors and known molecular data will be useful to determine if tall cell morphology is predictive of recurrence. Of note, on univariate analysis, PTCs without an available subtype designation had a decreased risk of recurrence relative to classic PTCs (Table 5); this finding is likely explained by the fact that the non-subtyped tumors were those ≤ 1 cm in which morphological subtype was not assigned at the time of diagnosis in light of historic designation as “microcarcinoma” subtype. PTCs with extensive follicular growth, which have significantly lower expression of *BRAF* p.V600E (Table 2), did not have a lower risk of recurrence in this study (Table 6), further supporting this study’s finding that *BRAF* p.V600E is not a risk factor for PTC recurrence.

Given that presence of *BRAF* p.V600E was not found to be a risk factor for PTC recurrence on multivariate analysis, this study sought to identify other tumor characteristics associated with recurrence. Larger tumor size was associated with increased risk of recurrence on univariate analysis (Table 5). However, on multivariate analysis, tumor size did not remain associated with recurrence. A likely confounding factor was nodal disease, because most tumors ≤ 2 cm had no nodal disease burden while most tumors > 2 cm had large volume nodal disease burden. Given that ATA guidelines specify that having no involved lymph node (LN) metastases (N0) or small volume LN metastases are low risk features for recurrence [22], this study examined nodal disease burden (based on pathologic examination) as a risk factor for recurrence. On multivariate analysis, nodal disease burden remained significantly associated with recurrence, with large volume nodal disease burden (> 5 involved LNs and > 0.2 cm nodal deposit) conferring a 3.37 times risk for PTC recurrence relative to tumors with no nodal disease burden on pathology (*p* = 0.004) (Table 6). A minority of patients were male sex (30%) (Table 1), and on multivariate analysis, male sex conferred a hazard ratio of 2.29 for recurrence (*p* = 0.009) (Table 6). Male sex is not currently included in the ATA risk stratification system [22], and whether male sex is a risk factor for recurrence is controversial; however, several studies argue recurrence is higher in males [49–51]. Almost half (47%) of PTCs in this cohort were multifocal (Table 1), and although not significant, on multivariate analysis, tumor multifocality conferred a 1.92 times higher risk of recurrence (*p* = 0.053) (Table 6). In this data set, 9% of patients (*n* = 26) had extrathyroidal extension (Table 1), and on multivariate analysis, although not significant, extrathyroidal extension conferred a 2.09 times higher risk of recurrence (*p* = 0.056) (Table 6).

Limitations to this study include: it is a single center retrospective study at a specialized referral center, which can introduce selection bias and affect the generalizability of the results. Molecular testing to assess for the presence of aggressive mutations was not uniformly performed, and therefore, molecular status aside from *BRAF* p.V600E was not able to be evaluated. Of note, 11/16 *BRAF* p.V600E positive patients and 0/13 *BRAF* p.V600E negative patients had either *TERT* promoter or *TP53* mutation on next generation sequencing. In light of few sequenced cases and selection bias for sequencing of aggressive cases, meaningful conclusions cannot be made based on presence or absence of high-risk mutations. Also, *BRAF* p.V600E status was determined for most patients based on VE1 immunostain. Therefore, additional false negatives or false positives would not be accounted for. However, significant data shifts are unlikely given the high sensitivity (99%, 95% CI 0.98–1.00) and specificity (84%, 95% CI 0.72–0.91) of VE1 IHC [29]. Of note, the one false negative tumor on IHC included in this study (Supplemental Fig. 1) was not decalcified, and thus, decalcification does not explain the false negative result. Furthermore, the variant allele frequency for BRAF p.V600E in this case was 29%. Repeat immunostain (for the purposes of this project) with stronger dilution yielded a positive result. Zhang et al. argue that BRAF VE1 staining intensity in PTCs is greater with longer incubation time, and that longer incubation time can reduce the number of false negatives on IHC [52]. Thus, it is it is likely that longer incubation times or strengthening of dilution could improve detection of mutant protein in tissue. Further large prospective studies are needed to assess the weight that clinicopathological risk factors (including features such as extranodal extension, not evaluated in this paper), have on recurrence in order to create a more comprehensive predictive model.

## Conclusions

Overall, the results of this study suggest that large volume nodal disease burden and male sex, and possibly tall cell morphology, tumor multifocality, and gross extrathyroidal extension are risk factors for recurrence that should be considered when determining treatment for PTC. However, presence of *BRAF* p.V600E (79% of PTCs) is not a risk factor for recurrence and consideration should be given to minimizing its importance in clinical decision-making.

## Supplementary Information

Below is the link to the electronic supplementary material.

Supplementary file1 (DOCX 279 KB)

## Data Availability

No datasets were generated or analysed during the current study.

## Declarations

### Research Involving Humans

Consent to participate was waived by the Institutional Board Review committee.
