# Supplementary material for: Prevalence and Clinical Impact of BRAF p.V600E Mutation in Papillary Thyroid Carcinoma
Source: Endocr Pathol. 2025 Apr 16;36(1):13. doi: 10.1007/s12022-025-09859-y (PMC12003545; doi:10.1007/s12022-025-09859-y)
Supplement: Supplementary file 1 — Supplementary file1 (DOCX 279 KB) [file 12022_2025_9859_MOESM1_ESM.docx]

**Article title:** Prevalence and Clinical Impact of *BRAF* p.V600E Mutation in Papillary Thyroid Carcinoma

**Journal Name:** Endocrine Pathology

**Corresponding author:** Nicole A. Cipriani, MD ([Nicole.Cipriani@bsd.uchicago.edu](mailto:Nicole.Cipriani@bsd.uchicago.edu))

**SUPPLEMENTAL TABLES AND FIGURES**


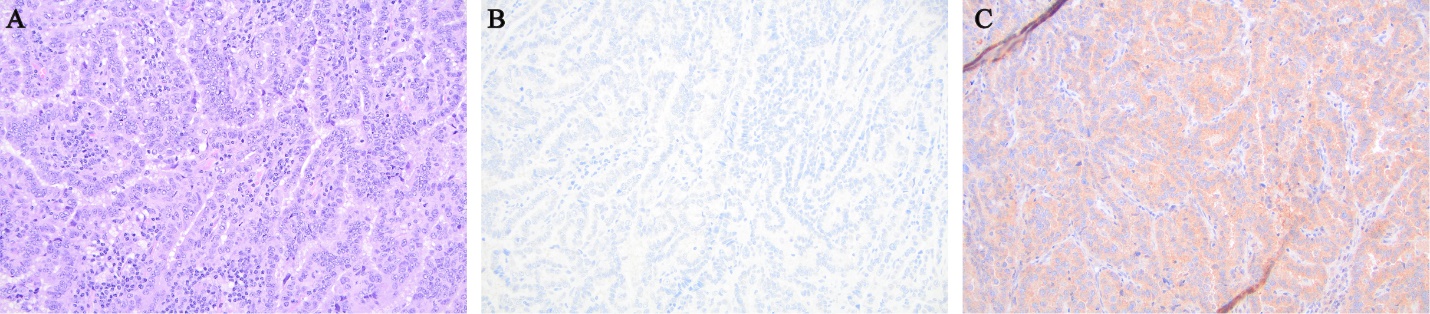


**Supplemental Figure 1.** Hematoxylin and eosin (H&E) stain **(A)** and initial BRAF p.V600E immunohistochemistry (IHC) (VE1 clone) **(B)** of the one papillary thyroid carcinoma (PTC) that was a false negative on IHC. Given molecular analysis demonstrated presence of *BRAF* p.V600E mutation, this patient was deemed *BRAF* p.V600E positive in the final analysis. Repeat immunostain (for purposes of this project) with stronger dilution is shown in **(C)**.

**Supplemental Table 1.** Additional demographics and clinicopathological characteristics, stratified by tumor subtype

|  | **Classic (n=185)** | **EFG (n=53)** | **Tall (n=35)** | **Not available (n=28)** | **p-value** |
| --- | --- | --- | --- | --- | --- |
| **Size in cm** |  |  |  |  | **< 0.001** |
| ≤1 cm | 42 (23%) | 11 (21%) | 2 (6%) | 28 (100%) |  |
| >1 and ≤2 cm | 75 (41%) | 23 (43%) | 12 (34%) | 0 (0%) |  |
| >2 and ≤4 cm | 52 (28%) | 16 (30%) | 15 (43%) | 0 (0%) |  |
| >4 cm | 16 (9%) | 3 (6%) | 6 (17%) | 0 (0%) |  |
| **Nodal disease burden on pathology** |  |  |  |  | **0.002** |
| None | 73 (39%) | 29 (55%) | 15 (43%) | 22 (79%) |  |
| Small volume | 23 (12%) | 4 (8%) | 1 (3%) | 3 (11%) |  |
| Large volume | 89 (48%) | 20 (38%) | 19 (54%) | 3 (11%) |  |
| **Multifocal** | 90 (49%) | 24 (45%) | 17 (49%) | 11 (39%) | 0.81 |
| **Extrathyroidal extension** | 14 (8%) | 4 (8%) | 8 (23%) | 0 (0%) | **0.008** |

**Supplemental Table 2.** Additional demographics and clinicopathological characteristics, stratified by primary tumor size in cm

|  | **≤1 cm (n=83)** | **>1 and ≤2 cm (n=110)** | **>2 and ≤4 cm (n=83)** | **>4 cm (n=25)** | **p-value** |
| --- | --- | --- | --- | --- | --- |
| **Subtype** |  |  |  |  | **< 0.001** |
| Classic | 42 (51%) | 75 (68%) | 52 (63%) | 16 (64%) |  |
| EFG | 11 (13%) | 23 (21%) | 16 (19%) | 3 (12%) |  |
| Tall | 2 (2%) | 12 (11%) | 15 (18%) | 6 (24%) |  |
| Not available | 28 (34%) | 0 (0%) | 0 (0%) | 0 (0%) |  |
| **Nodal disease burden on pathology** |  |  |  |  | **< 0.001** |
| Neither | 51 (61%) | 55 (50%) | 29 (35%) | 4 (16%) |  |
| Small volume | 11 (13%) | 8 (7%) | 9 (11%) | 3 (12%) |  |
| Large volume | 21 (25%) | 47 (43%) | 45 (54%) | 18 (72%) |  |
| **Multifocal** | 39 (47%) | 53 (48%) | 37 (45%) | 13 (52%) | 0.918 |
| **Extrathyroidal extension** | 0 (0%) | 9 (8%) | 10 (12%) | 7 (28%) | **< 0.001** |

**Supplemental Table 3.** Additional demographics and clinicopathological characteristics, stratified by nodal disease burden on final pathology

|  | **Neither (n=139)** | **Small volume (n=31)** | **Large volume (n=131)** | **p-value** |
| --- | --- | --- | --- | --- |
| **Subtype** |  |  |  | **0.002** |
| Classic | 73 (53%) | 23 (74%) | 89 (68%) |  |
| EFG | 22 (16%) | 3 (10%) | 3 (2%) |  |
| Tall | 29 (21%) | 4 (13%) | 20 (15%) |  |
| Not available | 15 (11%) | 1 (3%) | 19 (15%) |  |
| **Size in cm** |  |  |  | **< 0.001** |
| ≤1 cm | 51 (37%) | 11 (35%) | 21 (16%) |  |
| >1 and ≤2 cm | 55 (40%) | 8 (26%) | 47 (36%) |  |
| >2 and ≤4 cm | 29 (21%) | 9 (29%) | 45 (34%) |  |
| >4 cm | 4 (3%) | 3 (10%) | 18 (14%) |  |
| **Multifocal** | 55 (40%) | 20 (65%) | 67 (51%) | **0.02** |
| **Extrathyroidal extension** | 6 (4%) | 2 (6%) | 18 (14%) | **0.02** |

**Supplemental Table 4.** Clinical outcomes in tumors >1 cm, stratified by BRAF p.V600E status

|  | **BRAF negative (n=45)** | **BRAF positive (n=173)** | **All (n=218)** | **p-value** |
| --- | --- | --- | --- | --- |
| **Recurrence within 10 years** | 7 (16%) | 33 (19%) | 40 (18%) | 0.587 |
| **Distant metastasis** | 1 (2%) | 8 (5%) | 9 (4%) | 0.471 |
| **Death** | 1 (2%) | 2 (1%) | 3 (1%) | 0.586 |

**Supplemental Table 5.** Clinical outcomes in tumors >2 and ≤4 cm, stratified by BRAF p.V600E status

|  | **BRAF negative (n=18)** | **BRAF positive (n=65)** | **All (n=83)** | **p-value** |
| --- | --- | --- | --- | --- |
| **Recurrence within 10 years** | 2 (11%) | 14 (22%) | 16 (19%) | 0.321 |
| **Distant metastasis** | 0 (0%) | 2 (3%) | 2 (2%) | 0.451 |
| **Death** | 1 (6%) | 0 (0%) | 1 (1%) | 0.056 |
